# Supplementary figures and images for: Identification of Resveratrol as Bioactive Compound of Propolis from Western Romania and Characterization of Phenolic Profile and Antioxidant Activity of Ethanolic Extracts
Source: Molecules. 2019 Sep 16;24(18):3368. doi: 10.3390/molecules24183368 (PMC6766919; doi:10.3390/molecules24183368)

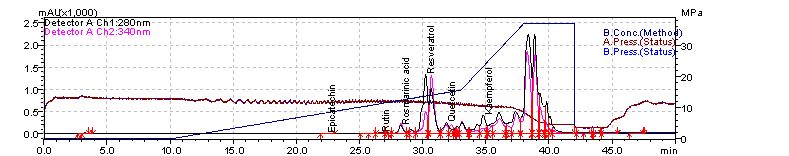

Supplement: Supplementary file 1 [file molecules-24-03368-s001.zip › P7.jpg]

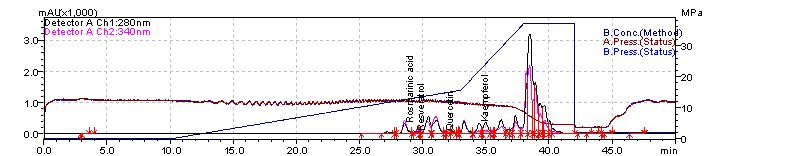

Supplement: Supplementary file 1 [file molecules-24-03368-s001.zip › P8.jpg]

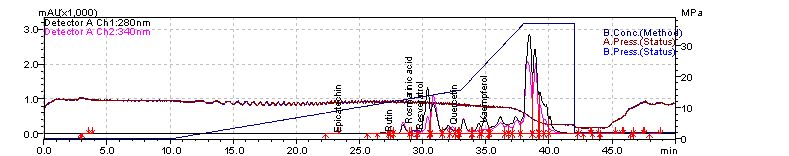

Supplement: Supplementary file 1 [file molecules-24-03368-s001.zip › P1.jpg]

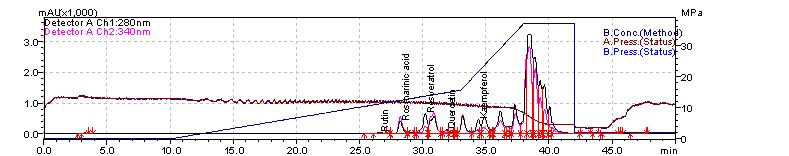

Supplement: Supplementary file 1 [file molecules-24-03368-s001.zip › P2.jpg]

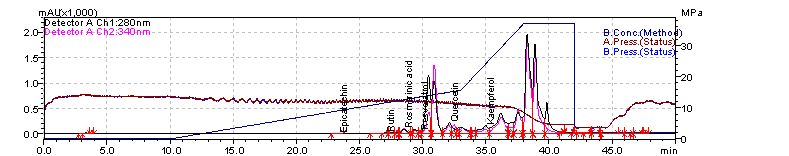

Supplement: Supplementary file 1 [file molecules-24-03368-s001.zip › P3.jpg]

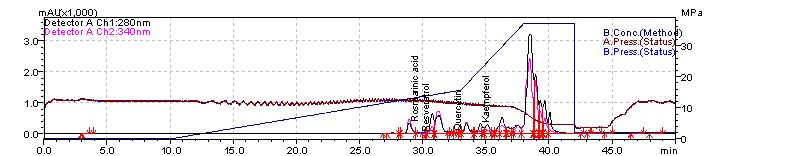

Supplement: Supplementary file 1 [file molecules-24-03368-s001.zip › P4.jpg]

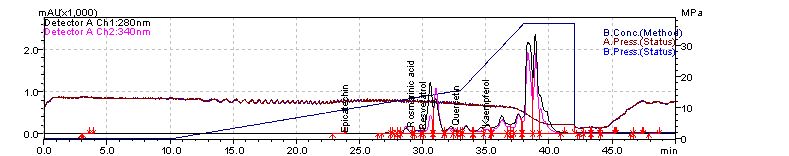

Supplement: Supplementary file 1 [file molecules-24-03368-s001.zip › P5.jpg]

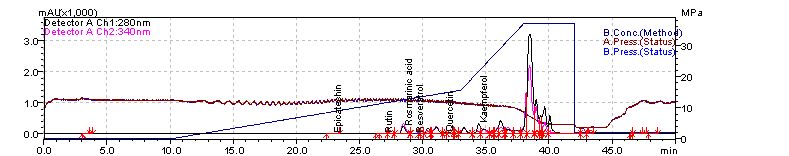

Supplement: Supplementary file 1 [file molecules-24-03368-s001.zip › P6.jpg]
